# Supplementary material for: Dependence of Skin-Electrode Contact Impedance on Material and Skin Hydration
Source: Sensors (Basel). 2022 Nov 4;22(21):8510. doi: 10.3390/s22218510 (PMC9656728; doi:10.3390/s22218510)
Supplement: Supplementary file 1 [file sensors-22-08510-s001.zip › sensors-1930644-supplementary.pdf]

Supplementary

Dependence of Skin-Electrode Contact Impedance on Material and Skin Hydration

Krittika Goyal [1,*], David A. Borkholder [1] and Steven W. Day [2]

[1] Department of Microsystems Engineering, Rochester Institute of Technology, Rochester, NY, USA
[2] Department of Biomedical Engineering, Rochester Institute of Technology, Rochester, NY, USA
* Correspondence: Krittika.Goyal@mail.rit.edu; Tel.: +1 585 214 9251

Table S1. Impedance model fitting parameters obtained for impedance response across SS electrodes of 4 cm^2 and 9 cm^2 area electrodes on a dry phantom. R_d and R_{sc} represent the resistance of the deeper tissue layer and stratum corneum respectively. Constant phase element CPE_{sc} and exponent α represent stratum corneum capacitance and CPE_c and exponent β represent contact capacitance. Values in the Table are shown as computed ± error.

Electrode Area (SS and dry)	R_d (kΩ)	R_{sc} (MΩ)	CPE_{sc} (nSs$^{\alpha}$)	α	CPE_c (µSs$^{\beta}$)	β
4 cm^2	1.77 ± 0.00	1.62 ± 0.00	182 ± 3.77	0.9 ± 0.005	1.02 ± 0.03	0.56 ± 0.003
9 cm2	1.75 ± 0.03	0.70 ± 0.04	500 ± 10.8	0.8 ± 0.011	2.02 ± 0.42	0.56 ± 0.010

Table S2. Impedance model fitting parameters obtained for impedance response across SS and Ti on a dry and hydrated phantom. Impedance normalized to the area is shown. R_d and R_{sc} represent the resistance of the deeper tissue layer and stratum corneum respectively. Constant phase element CPE_{sc} and exponent α represent stratum corneum capacitance and CPE_c and exponent β represent contact capacitance. Values in the Table are shown as computed ± error.

Electrode Material (Phantom Hydration status)	R_d (kΩcm^2)	R_{sc} (MΩcm^2)	CPE_{sc} (nSs$^{\alpha}$cm^{-2})	α	CPE_c (nSs$^{\beta}$cm^{-2})	β
Ti (dry)	9.3 ± 0.20	6.4 ± 0.36	47.7 ± 1.08	0.9 ± 0.01	424 ± 45.6	0.50 ± 0.012
SS (dry)	9.3 ± 0.00	6.4 ± 0.00	47.7 ± 0.00	0.9 ± 0.00	170 ± 2.75	0.56 ± 0.002
Ti (hydrated)	9.3 ± 0.00	3.0 ± 0.00	200 ± 0.00	0.9 ± 0.00	652 ± 11.1	0.52 ± 0.002
SS(hydrated)	9.3 ± 0.00	3.0 ± 0.00	200 ± 0.00	0.9 ± 0.00	327± 4.65	0.59 ± 0.002

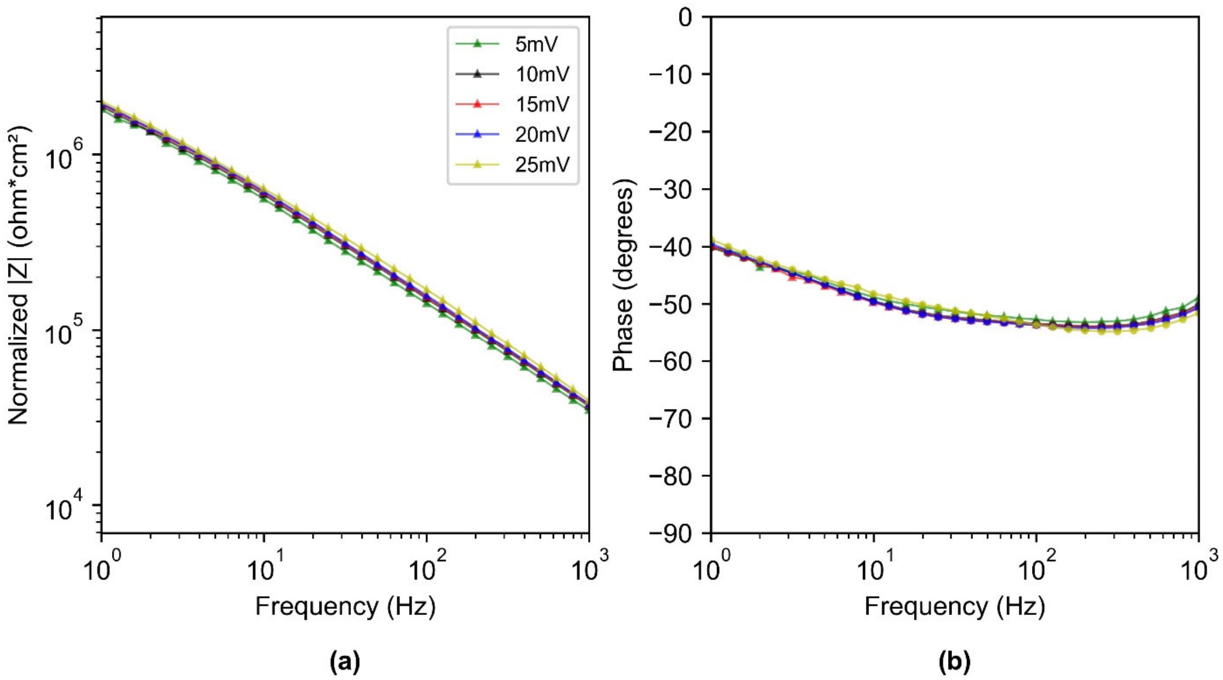

Figure S1. Bode plot depicting the a) normalized impedance magnitude with respect to area and b) phase for SS (9 cm^2) on a hydrated phantom obtained with the excitation voltage of 5 mV, 10 mV, 15 mV, 20 mV and 25 mV.
